# JAK-STAT pathway, type I/II cytokines, and new potential therapeutic strategy for autoimmune bullous diseases: update on pemphigus vulgaris and bullous pemphigoid

**DOI:** 10.3389/fimmu.2025.1563286

**Published:** 2025-04-08

**Authors:** Xiaoying Lin, Xiang Li, Zhifang Zhai, Mingwang Zhang

**Affiliations:** Department of Dermatology, The First Affiliated Hospital, Army Medical University, Chongqing, China

**Keywords:** autoimmune bullous diseases, pemphigus vulgaris, bullous pemphigoid, JAK inhibitors, cytokines, JAK-STAT pathway

## Abstract

Autoimmune Bullous Diseases (AIBDs), characterized by the formation of blisters due to autoantibodies targeting structural proteins, pose significant therapeutic challenges. Current treatments, often involving glucocorticoids or traditional immunosuppressants, are limited by their non-specificity and side effects. Cytokines play a pivotal role in AIBDs pathogenesis by driving inflammation and immune responses. The JAK-STAT pathway is central to the biological effects of various type I and II cytokines, making it an attractive therapeutic target. Preliminary reports suggest that JAK inhibitors may be a promising approach in PV and BP, but further clinical validation is required. In AIBDs, particularly bullous pemphigoid (BP) and pemphigus vulgaris (PV), JAK inhibitors have shown promise in modulating pathogenic cytokine signaling. However, the safety and selectivity of JAK inhibitors remain critical considerations, with the potential for adverse effects and the need for tailored treatment strategies. This review explores the role of cytokines and the JAK-STAT pathway in BP and PV, evaluating the therapeutic potential and challenges associated with JAK inhibitors in managing these complex disorders.

## 1 Introduction

Autoimmune bullous diseases (AIBDs) encompass a spectrum of conditions characterized by the formation of vesicles, blisters, erosions, excoriations, and erythemas on the skin and/or mucosal membranes, which can lead to serious complications and even death due to superinfections, loss of body fluids and severely limited food intake (1). AIBDs can be broadly classified into two categories, pemphigus (intraepidermal blistering) and pemphigoid (subepidermal blistering), depending on the location of the blisters (2, 3). Pemphigus disorders are caused by autoantibodies directed against the desmosomal proteins desmoglein (Dsg) 1 and 3 and mainly include pemphigus vulgaris (PV), pemphigus foliaceus (PF), paraneoplastic pemphigus (PNP) and immunoglobulin A (IgA) pemphigus (4–6). In contrast, pemphigoid disorders are triggered by autoantibodies targeting various autoantigens within or underneath the basement membrane zone (BMZ), such as BP180, BP230, laminins and type VII collagen. This group mainly includes bullous pemphigoid (BP), linear IgA bullous dermatosis, dermatitis herpetiformis (DH), mucous membrane pemphigoid (MMP), anti-p200 pemphigoid (targeted laminin γ1) (7),epidermolysis bullosa acquisita (EBA), bullous systemic lupus erythematosus (BSLE) and herpes gestationis (8).

The treatment of AIBDs is predominantly involve glucocorticoids and immunosuppressive agents, which have serious adverse effects with prolonged use and whose efficacy is highly heterogeneous across patients (9, 10). In recent years, more precisely immunotherapy that targets cytokines or pivotal proteins extracellularly, such as rituximab against CD20, omalizumab against IgE, and dupilumab against IL-4Rα, are increasingly being utilized for the treatment of AIBDs that is unresponsive to glucocorticoids or immunosuppressants, and have demonstrated promising outcomes (11, 12). While these therapies offer benefits, some patients still exhibit unresponsive, which leads to mortality rates for patients with AIBDs remaining significantly higher as compared with the general population (13, 14). Hence, new strategies that target intracellular pathways activated by cytokines warrant consideration in the future.

Cytokines are a group of structurally distinct secreted proteins that bind to cellular receptors belonging to at least seven superfamilies which exert their biological effects through very different signaling pathways. Among them, Janus kinase (JAK) and signal transducer and activator of transcription (STAT) pathways are required for the effective responses of innate and adaptive immune by regulating the signaling cascade of type I and type II cytokines (15). Dysfunction of the JAK-STAT signaling pathway has been linked to a range of inflammatory and autoimmune diseases, and JAK inhibitors have emerged as a promising therapeutic approach (16). Indeed, over the past decade, several small-molecule JAK inhibitors have been approved for the treatment of immune-mediated diseases such as rheumatoid arthritis (RA), psoriatic arthritis, ankylosing spondylitis, atopic dermatitis (AD), alopecia areata (AA) and others (17). In contrast to JAK inhibitors, until now, STAT inhibitors have not been marketed due to the structural peculiarities and functional complexities of STAT, which are still in the clinical research stage and are primarily concentrated in the field of cancer (18). This review will elucidate the present recognition of JAK-STAT signaling and the pathway-dependent type I/II cytokines in immune homeostasis. Furthermore, we describe the involvement of the cytokine network and JAK-STAT pathway in the pathogenesis of PV and BP, and discuss advancements in JAK-targeted therapeutics for these conditions. Finally, we explore the safety and selectivity in the clinical implementation of the JAK inhibitors.

## 2 Overview of JAK-STAT signaling pathway

**Figure 1:**
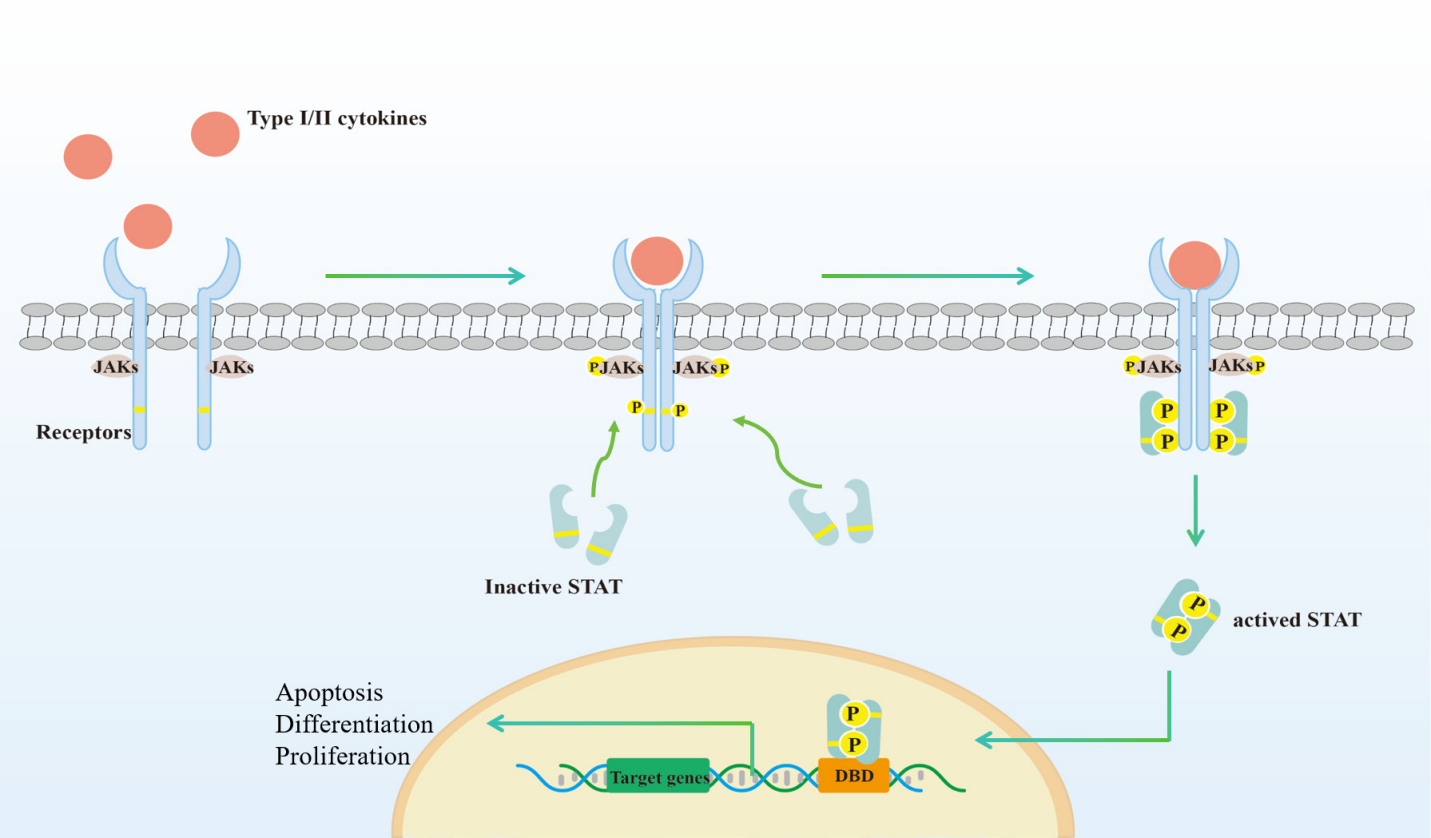
Activation of the canonical JAK-STAT signaling pathways. (1) Type I//II cytokines act through receptors associated with JAK. The receptors comprise at least two chains, each linked to a specific JAK; (2) Binding of ligand dimerizes the receptor, resulting in phosphorylation and activation of JAK for each other, which then phosphorylates the receptor. The STAT family has an N-terminal structural domain that allows STAT to form inactive dimers; (3) STAT bind to the phosphorylated receptor, which in turn phosphorylates by JAK; (4) STAT detach from the receptor to form activated dimers; (5) STAT dimers enter the nucleus, bind to DNA binding domain (DBD) and regulate transcription, which is involved in cell proliferation, differentiation and apoptosis. Created with Adobe Illustrator2023.

The JAK family comprises highly conserved mammalian protein non-receptor tyrosine kinases, which include JAK1, JAK2, JAK3 and tyrosine kinase 2 (TYK2). JAKs are ubiquitously expressed, with the exception of JAK3, which is predominantly found in immune cells (19). The canonical JAK/STAT signaling pathway is as follows (**Figure 1**): JAKs are noncovalently associated with the cytoplasmic domain of the cytokine receptors’ signaling chain. The binding of type I and II cytokines to the receptor induces dimerization or aggregation of the receptor’s signaling chains. This process brings JAKs into close proximity, leading to autophosphorylation or transphosphorylation. The activated JAKs then phosphorylate the associated receptor on specific tyrosine residues. The phosphorylated receptor, in turn, recruits a STAT protein (STAT1, STAT2, STAT3, STAT4, STAT5A, STAT5B, or STAT6), which is also phosphorylated by the activated JAK on its conserved C-terminal tyrosine residue. This event triggers the STAT protein’s homodimerization or heterodimerization and its subsequent translocation to the nucleus (20), where it functions as a transcription factor, regulating the expression of genes related to immune cell growth, proliferation, differentiation, and apoptosis (21–23). In addition to canonical signaling, studies have reported that JAK-STAT is also engaged in noncanonical signal transduction, which is relatively more complicated (24, 25). For example, unphosphorylated STAT3 could also bind to DNA and act as a transcriptional activator (26).

The JAK-STAT pathway is closely related to immune homeostasis and the development of autoimmune diseases and is thus subject to intricate regulation. This regulatory network includes three main negative regulators: a variety of protein tyrosine phosphatases (27–29), suppressors of cytokine signaling (SOCSs) (30–32), and protein inhibitors of activated STATs (PIASs) (33–35), as well as multiple positive regulators (e.g., cooperating transcription factors (36)). Mutations in genes encoding JAK or STAT proteins are associated with a variety of immune-related diseases. For example, inactivating mutations in JAK3 can cause severe combined immunodeficiency (SCID) (37, 38), while loss-of-function mutations in TYK2 result in milder immunodeficiency (39). Similarly, gain-of-function mutations in STAT1 are linked to a spectrum of infectious and autoimmune diseases (40), whereas loss-of-function in STAT3 leads to hyper-IgE syndrome, characterized by recurrent skin and pulmonary infections, elevated IgE levels, and chronic eczematous dermatitis (41). Thus, a substantial body of evidence indicates that abnormal activation of the JAK-STAT pathway is pivotal in the pathogenesis of human immune diseases.

## 3 Overview of type I and type II cytokines

Cytokines are classified into superfamilies based on the shared structural features of their cognate receptors. More than 50 cytokines, including interleukins (ILs), interferons (IFNs), hormones, and colony-stimulating factors (CSFs), are members of the type I/II cytokine families and signal via the JAK-STAT pathway. These cytokines can be further subdivided into groups based on their shared receptor subunits (**Table 1**).

**Table 1:** Type I/type II cytokines, their receptors, JAK kinases, STAT proteins, and function.

Cytokine	Receptors	JAKs	STATs	Actions in immune cells
Type I cytokine				
Common γc family				
IL-2	IL-2Rα, IL-2Rβ, γc	JAK1, JAK3	STAT5, STAT1, STAT3	T-cell proliferation and differentiation, B and NK cells activation, Treg maintenance, Induces AICD of T cells
IL-4	IL-4Rα, γc	JAK1, JAK3	STAT6, STAT5	B cell activation, lg isotype switching, Th2 and Th9 cell differentiation, inhibits Treg proliferation
IL-4/IL-13*	IL-4Rα, IL-13Rα1	JAK1, TYK2/JAK2	STAT6, STAT5	B-cell maturation and differentiation, IgE isotype switching, inhibits macrophage activity and Th1 cell
IL-7	IL-7Rα, γc	JAK1, JAK3	STAT5, STAT1, STAT3	Growth of pre-B cells, maintain naïve, memory T cells and ILCs homeostasis, Treg development
TSLP*	IL-7Rα, TSLPR	JAK1, JAK2	STAT5, STAT1, STAT3	Stimulates hematopoietic cells and dendritic cells, induce Th2 responses
IL-9	IL-9Rα, γc	JAK1, JAK3	STAT5, STAT1, STAT3	Promotes mast cell growth, induces Th17 and ILC2 proliferation, enhances Treg function
IL-15	IL-15Rα, IL-2Rβ, γc	JAK1, JAK3	STAT5, STAT1, STAT3	Activation of NK cells, survival of CD8 memory T cells, mast cell growth factor
IL-21	IL-21Rα, γc	JAK1, JAK3	STAT3, STAT1, STAT5	Induces Ig production, B-cell differentiation and apoptosis, Th17 and Tfh differentiation, Antagonizes Th9 and Treg differentiation, promotes CD8 T cells proliferation and function
Common βc family				
IL-3	IL-3Rα, βc	JAK2, JAK1	STAT5	Growth and differentiation of CD34+ progenitor cells
IL-5	IL-5Rα, βc	JAK2, JAK1	STAT5	stimulation of eosinophils
GM-CSF	GMRα, βc	JAK2, JAK1	STAT5	Growth and differentiation of dendritic cells, myelomonocyte progenitors and granulocytes
IL-6 family				
IL-6	IL-6Rα, gp130	JAK1, JAK2, TYK2	STAT3, STAT1	Acute phase protein production, induces Th17 subset and B-cell growth and differentiation, inhibits Treg differentiation
IL-11	IL-11Rα, gp130	JAK1, JAK2, TYK2	STAT3, STAT1	Hematopoiesis, suppress macrophage activity, Th2 and Th17 cell differentiation, B cell differentiation and IgG production
IL-27	WSX-1, gp130	JAK1, JAK2, TYK2	STAT1, STAT3	Induces Th1 cell differentiation, promotes Treg cell differentiation and IL-10 production, inhibits Th2 and Th17 response
IL-31^*^	OSMRβ, IL-31RA	JAK1, JAK2, TYK2	STAT3, STAT5, STAT1	Hematopoiesis, induces Th2 response
IL-35	IL-12Rβ2, gp130	JAK1, JAK2, TYK2	STAT1, STAT4	Augments Treg and Breg proliferate and activity
IL-39	IL-23R, gp130	JAK1, JAK2, TYK2	STAT1, STAT3	Promotes neutrophils differentiation and development
OSM	OSMRβ/LIFRβ, gp130	JAK1, JAK2, TYK2	STAT5, STAT1, STAT3	Hematopoiesis
LIF	LIFRβ, gp130	JAK1, JAK2, TYK2	STAT3, STAT1	Hematopoiesis, promotes Treg cell differentiation, suppresses Th17 cell differentiation
CT-1	LIFRβ, gp130	JAK1, JAK2, TYK2	STAT3, STAT1	Cause apoptosis of inflammatory cells
CLCF1	CNTFRα, LIFRβ, gp130	JAK1, JAK2, TYK2	STAT3, STAT1	Hematopoiesis and immune cell function
CNTF	CNTFRα/IL-6Rα, LIFRβ, gp130	JAK1, JAK2, TYK2	STAT3, STAT1	
IL-12 family				
IL-12	IL-12Rβ1, IL-12Rβ2	JAK2, TYK2	STAT4, STAT1, STAT3, STAT5	Facilitates Th1 cells differentiation, Activates NK cells
IL-23	IL-12Rβ1, IL-23R	JAK2, TYK2	STAT3, STAT1, STAT4, STAT5	Induces Th17 cells development
Type II cytokine				
IFN family				
Type I	IFNAR1, IFNAR2	TYK2, JAK1	STAT1, STAT2, STAT3, STAT5	Promotes or inhibits T cell and B cell proliferation, differentiation and activation
Type II	IFNGR1, IFNGR2	JAK1, JAK2	STAT1, STAT3	Promotes macrophage activation, Th1 cell differentiation and Ig class switching, suppresses Th2 and Th17 cell response
Type III	IFNLR1, IL10Rβ	JAK1, TYK2	STAT1, STAT2, STAT3, STAT5	suppress neutrophil recruitment and activity, promote Th1 cell response and CD8+ T cell activity
IL-10 family				
IL-10	IL-10RA, IL-10RB	JAK1, TYK2	STAT3, STAT1, STAT5	Inhibits APCs, memory Th17 and Th2 cells, promotes Tregs function and B cell differentiation and isotype switching
IL-19	IL-20RA, IL-20RB	JAK1, TYK2	STAT3, STAT1, STAT5	Promotes Th2 cell response and macrophages to M2 type
IL-20	IL-20RA/IL-22RA1, IL-20RB	JAK1, TYK2	STAT3, STAT1, STAT5	Promotes DC maturation
IL-22	IL-22RA1, IL-10RB	JAK1, TYK2	STAT3, STAT1, STAT5	Regulates neutrophil recruitment
IL-24	IL-20RA/IL-22RA1, IL-20RB	JAK1, TYK2	STAT3, STAT1, STAT5	Inhibits plasma cell differentiation
IL-26	IL-20RA, IL-10RB	JAK1, TYK2	STAT3, STAT1, STAT5	Priming of neutrophils, NK cells and pDCs

*The receptors for IL-4/IL-13 and IL-31 are not γc and gp130, respectively. However, they are closely related to γc and gp130, share structural similarities with them, and can exert biological effects through the JAK-STAT pathway.

### 3.1 Type I cytokines in immunoregulation

#### 3.1.1 The common γ chain (γc) family

The common γc family cytokines include IL-2, IL-4, IL-7, IL-9, IL-15, and IL-21. A mutation in the gene encoding the protein γc has been associated with SCID in humans, indicating that these cytokines play a fundamental role in the development of the immune system. The inaugural member of this family identified was IL-2, originally discovered as a T cell growth factor in 1976 (42). IL-2 transmits signals through JAK1 and JAK3, which covalently bind to the IL-2Rβ and γc, respectively (43), and predominantly recruit and activate STAT5A and STAT5B, as well as to a lesser extent, STAT3 and STAT1 (44). IL-2 has been shown to exhibit pleiotropic actions that are wide-ranging and significant (45). Specifically, IL-2 is known to promote the growth and differentiation of B cells (46), augment the proliferation of natural killer (NK) cells while enhancing their cytotoxicity (47). Additionally, IL-2 is essential for the development and expansion of T regulatory (Treg) cells (48), and for activation-induced cell death of T cells (49), which helps mediate tolerance and limit inappropriate immune responses. Furthermore, it aids in the differentiation of naïve CD4^+^ T cells into T helper 1 (Th1) (50), Th2 (51) and Th9 cells (52), while inhibiting the differentiation of Th17 (53) and T follicular helper (Tfh) cells (54).

IL-4 was originally discovered as a cytokine that stimulates B cell activation and increases the production of immunoglobulin class switch, resulting in elevated levels of IgG1 and IgE (55, 56). Further studies have shown that IL-4 plays a key role in driving Th2 responses (57) and promoting Th9 differentiation, while simultaneously suppressing the generation of transforming growth factor-β (TGF-β)-induced Foxp3^+^ Treg cells (58). IL-4 signals through two types of receptors: type I and type II. Type I IL-4R, expressed on hematopoietic cells, consists of IL-4Rα and γc subunits (59, 60) and is linked to JAK1 and JAK3, respectively (61). The type II IL-4R, composed of the IL-4Rα and IL-13Rα1, is primarily expressed on non-hematopoietic cells and is linked to JAK1 and TYK2/JAK2, which is also the functional receptor for IL-13 (62, 63). Both type I and type II IL-4 receptors mainly activate STAT6 and, to a lesser extent, STAT5 (64, 65).

IL-7 was first identified as a stromal-cell-derived factor that facilitates the growth of pre-B cells (66, 67). Apart from its role in pre-B cell development, IL-7 and its receptor are crucial for maintaining the development of T cells and innate lymphoid cells (ILCs) and protecting the survival of long-term memory T-cell (68, 69). Additionally, IL-7 contributes to the development of Treg cells (70). The IL-7 receptor is comprised of two subunits, IL-7Rα and γc (71, 72), which bind to JAK1 and JAK3, respectively, and initiate downstream signaling, most notably STAT5A and STAT5B (73). IL-7Rα is also a functional component of the receptor for thymic stromal lymphopoietin (TSLP), along with another component called TSLPR (74). TSLP mediates STAT5, STAT3 and STAT1 phosphorylation via kinases JAK1 and JAK2 (75), and is crucially important for stimulating hematopoietic cells and mediating type 2 immunity (76).

IL-9 was initially identified in mice as a cytokine that promotes the proliferation of T cells (77). Upon binding to its heterodimeric receptor composed of the IL-9Rα and γc, IL-9 triggers the cross-phosphorylation of JAK1 and JAK3, leading to the activation of STAT5, STAT3, and STAT1 (78, 79). Beyond its role in T cell growth, IL-9 acts as a growth factor for bone marrow mast cells and promotes the secretion of several cytokines, including IL-13 (80, 81). In addition, IL-9 facilitates the expansion of Th17 cell and ILC2 populations while enhancing the regulatory function of Treg cells (82, 83).

IL-15 was initially identified as a growth factor for T-cells (84). IL-15 binds to a heterotrimeric receptor composed of IL-15Rα, IL-2Rβ, and γc (85). IL-15 binds to IL-15Rα on one cell and then trans-presents the cytokine to neighboring cells that express IL-2Rβ and γc, followed by activating the JAK1/JAK3 and STAT5 pathways (86), and plays a crucial role in the development of NK cells ^86^ and the maintenance of memory CD8^+^ T cells ^87^. Additionally, it has been found to act as a growth factor for mast cells (87).

IL-21 was first discovered as the ligand for an orphan type 1 cytokine receptor, which was found to bear a striking resemblance to IL-2Rβ (88, 89). Upon binding to its receptor, IL-21 stabilizes the complex between the IL-21Rα and the common γc (90). This event consequently activates JAK1 and JAK3, thereby facilitating the recruitment and activation of STAT proteins, with STAT3 being the predominant species, followed by STAT1 and STAT5 (91, 92). In the immune system, IL-21 has the ability to support the production of IgG1 and simultaneously repress the production of IgE (93). Additionally, IL-21 is capable of driving B cells toward plasma cell differentiation, while also promoting apoptosis of incompletely activated B cells (94, 95). Furthermore, IL-21 collaborates with IL-7 or IL-15 to expand CD8^+^ T cells (96) and contributes to the formation of CD8^+^ T cell memory (97). IL-21 also plays a role in the differentiation of Tfh (98) and Th17 cells (99), but inhibits the differentiation of Treg (100) and Th9 cells (52).

#### 3.1.2 The common β chain (βc) family

The βc family of cytokines, comprising IL-3, IL-5, and granulocyte-macrophage colony-stimulating factor (GM-CSF), was initially identified as CSFs in the hematopoietic system. However, they are now recognized as pleiotropic in the immune system (101, 102). The functional receptors for IL-3, IL-5, and GM-CSF are IL-3α paired with βc, IL-5α with βc, and GM-CSFα with βc, respectively (103). Upon binding to their specific receptor-alpha chains, the cytokine-alpha chain binary complexes dimerize with βc to form heterodimers. This process activates JAKs, primarily JAK2, which bind to the cytoplasmic tail of the βc chain, ultimately leading to the phosphorylation of STAT5A/B for downstream signaling (104).

βc cytokines possess the capability to influence various types of cells in the hematopoietic system. For instance, IL-3 is involved in the growth and differentiation of CD34^+^ progenitor cells into basophils and mast cells, DCs, eosinophils, and monocytes-macrophages (105, 106). Recent studies suggest that IL-3 modulates type 1 DC function to induce Th2 responses (107). IL-5, primarily responsible for stimulating eosinophils, is essential for eosinophilic inflammation (108, 109). GM-CSF acts on DCs, myelomonocyte progenitors, and granulocytes (110) while inhibiting CD34^+^ progenitor cell differentiation into lymphoid progenitors or type 2 DCs, as well as the terminal differentiation of mast cells (111, 112).

#### 3.1.3 The IL-6 family

The IL-6 family of cytokines encompasses a diverse group, including IL-6, IL-11, IL-27, IL-31, IL-35, IL-39, oncostatin M (OSM), leukemia inhibitory factor (LIF), ciliary neurotrophic factor (CNTF), cardiotrophin 1 (CT-1) and cardiotrophin-like cytokine factor 1 (CLCF1). The unifying feature of this family is their shared reliance on the gp130 receptor signaling subunit, which is pivotal for their classification (113).

The cytokines within the IL-6 family utilize several distinct receptor arrangements. IL-6 and IL-11 engage a specific non-signaling receptor subunit (IL-6Rα and IL-11Rα, respectively) in addition to the signal-transducing receptor gp130, forming either an IL-6–IL-6Rα–gp130 or IL-11–IL-11Rα–gp130 hexameric complex for signal transduction (114). Conversely, the receptor complexes for LIF, CT-1, OSM, IL-27, IL-35 and IL-39 comprise gp130 and a second signal transducing receptor subunit (LIFRβ, OSMRβ, WSX-1, IL-12Rβ2 and IL-23R, respectively), which share structural similarities with gp130 (115). The receptor for CNTF and CLCF1 comprises three individual receptor subunits, including a non-signaling receptor subunit CNTFRα and the heterodimer gp130 (LIFRβ and gp130) (116). The only cytokine exception to this “gp130 rule” is IL-31, which binds to a receptor complex containing OSMRβ and a unique gp130-like receptor chain, designated as IL-31RA (117). The cytokine receptor complexes within the IL-6 family transmit signals inside cells through the activation of JAK1, and to a lesser extent JAK2, or TYK2 (118, 119). These kinases are covalently bound to the cytoplasmic domains of their respective signal-transducing receptors. Upon activation, JAK proteins phosphorylate and thereby activate latent transcription factors, including STAT1, STAT3, and STAT5 (120, 121). The specific STAT proteins activated vary with the cytokine, correlating with the diverse biological functions of the IL-6 family cytokines. For example, IL-27 preferentially induces STAT1 activity, while IL-6 predominantly enhances STAT3 transcriptional activity (122). A summary of the specific immune functions of individual cytokines is presented in **Table 1**.

#### 3.1.4 The IL-12 family

The IL-12 cytokine family, comprising IL-12 and IL-23, shares structural similarities with the IL-6 family. These cytokines leverage orthologs of the gp130 receptor subunit to activate the JAK-STAT signaling pathway (123). IL-12 was first identified as a protein released by a specific human lymphoblastoid cell line that activates NK cells and T cells to produce IFNγ (124). IL-12 forms a complex with two different subunits, namely IL-12Rβ1 and IL-12Rβ2 (125). Upon binding, this complex activates JAK2 and TYK2, which subsequently recruit and phosphorylate STATs, mainly STAT4 (126). The phosphorylated STATs form dimers, translocate to the nucleus, and regulate gene transcription, playing a pivotal role in immune homeostasis. IL-12, a pro-inflammatory cytokine, drives the differentiation of CD4^+^ T cells into Th1-like cells and stimulates IFNγ production (127). Additionally, IL-12 can enhance the release of IFNγ and TNF in CD8^+^ T cells, NK cells, and ILC1s (128).

IL-23 was discovered a decade after the identification of IL-12 and was first characterized for its effects on memory T-cells but not naïve T-cells (129). The IL-23 receptor is composed of the IL-12Rβ1 and IL-23R chains. Similar to IL-12, the binding of IL-23 leads to the activation of JAK2 and TYK2, culminating in the phosphorylation of STATs, primarily STAT3 (130, 131). Research indicates that IL-23 is instrumental in promoting the expansion and maintenance of Th17 cells, without influencing their differentiation (132).

### 3.2 Type II cytokines in immunoregulation

#### 3.2.1 The IFN family

Since the discovery of the first IFN in 1957, based on its antiviral activity, more than 20 signaling molecules belonging to the IFN family have been identified (133). IFNs can be classified into three distinct classes of cytokines: type I IFNs, type II IFNs, and type III IFNs. The type I IFN family encompasses IFN-α (which can be further divided into 13 subtypes), IFN-β, IFN-δ, IFN-ϵ, IFN-κ, IFN-τ, and IFN-ω. All type I IFNs bind to a common receptor, composed of two subunits, IFNAR1 and IFNAR2, that interact with JAK1 and TYK2, respectively. Activation of JAKs rapidly phosphorylates STAT1, STAT2, STAT3, or STAT5, which then form homodimers or heterodimers, translocate to the nucleus, and initiate transcription (134). Beyond their direct antiviral effects, type I IFNs exhibit a range of immunomodulatory mechanisms. These include the promotion or inhibition of T-cell proliferation, differentiation, and apoptosis, contingent upon the timing of T cell receptor stimulation (135) and the duration of IFN exposure (135, 136). Type I IFNs promote B-cell activation and Ig class switching (137) but also inhibit B-cell growth and proliferation (138, 139), depending on different environments and antigens. These diverse biological effects may be mediated by the recruitment of different STATs (140).

In contrast to type I IFNs, type II IFNs consist of a single cytokine, IFN-γ (141). An IFN-γ homodimer binds to two IFNGR1 receptor chains and subsequently recruits two IFNGR2 chains to form a complex. The aggregation of its receptor components by IFN-γ leads to the phosphorylation of JAK1 and JAK2, which constitutively bind to IFNGR1 and IFNGR2, respectively. The activated JAKs further phosphorylate the STAT1 docking site in IFNGR1, followed by the recruitment and phosphorylation of STAT1. The phosphorylated STAT1 then dimerizes and translocates to the nucleus, where it regulates gene transcription (142). In addition to its antiviral activity, IFN-γ possesses pleiotropic immunologic functions, including the promotion of macrophage activation, enhancement of antigen presentation, induction of Th1 cell differentiation while concomitant inhibition of Th2 and Th17 cell responses, and promotion of Ig isotype switching (143).

Type III IFNs were discovered almost 50 years after the identification of type I IFNs and comprise four subtypes, IFN-λ1 (IL-29), IFN-λ2 (IL-28A), IFN-λ3 (IL-28B), and IFN-λ4 (144, 145). All type III IFN cytokines signal through a shared heterodimeric receptor, composed of IFNLR1 and IL10Rβ (146, 147). Like type I interferons, ligand binding activates JAK1 and TYK2, as well as downstream STATs transcription factors, primarily STAT1 and STAT2 (148). Although the downstream signaling pathways and transcriptional responses activated by type III IFNs are remarkably similar to those of type I IFNs, type III IFNs also display unique roles in the immune response. For example, type III IFNs protect epithelial cells from viral, bacterial, and fungal infections (149). In terms of immune cells, type III IFNs inhibit neutrophil recruitment and activity (150) and promote Th1 cell responses and CD8^+^ T cell activity (151).

#### 3.2.2 IL-10 family cytokine

The IL-10 family comprises IL-10 and five members of the IL-20 subfamily: IL-19, IL-20, IL-22, IL-24, and IL-26 (152). IL-10 is a cytokine that plays a crucial role in regulating the immune system. It signals through its heterodimeric receptor, which consists of two subunits: IL-10RA and IL-10RB (153, 154). The receptor complex associates with two JAKs, JAK1 and TYK2, respectively (155). Upon binding to the receptor, IL-10 primarily activates STAT3, although it can also activate STAT1 and STAT5 in specific cell types (156). This leads to suppressive effects on myeloid cells by inhibiting proinflammatory cytokines and antigen-presenting cells (APCs). Furthermore, IL-10 directly inhibits memory Th17 and Th2 cells, while promoting the survival and function of Tregs and the differentiation and isotype switching of B cell (157, 158).

The IL-20 subfamily of cytokines signals through various types of receptors. Specifically, IL-20RB, the common β chain, can form a functional heterodimeric receptor with either the IL-20RA, enabling binding of IL-19, IL-20, and IL-24, or with the IL-22RA1, which only allows signaling of IL-20 and IL-24. Conversely, IL-10RB, the other common β chain, heterodimerizes with either IL-22RA1 or IL-20RA to create a functional receptor for IL-22 or IL-26, respectively (159). All IL-20 subfamily cytokines transmit signals through the JAK-STAT pathway. Notably, all family members preferentially phosphorylate JAK1 and TYK2, which in turn primarily activate STAT3 (160). The IL-20 subfamily of cytokines possesses partially similar biological activities, such as promoting epidermal integrity and innate defense (161). However, each cytokine has distinctive functions. For instance, IL-19 is responsible for polarizing T-lymphocytes to Th2 type and macrophages to M2 type (162), whereas IL-20 is involved in the promotion of DC maturation (163). IL-20 has been found to regulate neutrophil recruitment (164) while IL-24 could stimulate peripheral blood mononuclear cell to produce pro-inflammatory cytokines and inhibit plasma cell differentiation (165, 166).

In addition to the aforementioned cytokines closely related to the JAK-STAT pathway, other cytokines, such as TNF-α, IL-1 or IL-17 family, etc., although not signaling directly through the JAK-STAT pathway, are influenced by JAK-STAT-dependent cytokines in terms of expression and biological function due to cytokine network effects (15). These further underscores the importance of the JAK-STAT pathway in immune homeostasis and immune-mediated diseases.

## 4 Cytokine and JAK-STAT pathway in AIBDs

### 4.1 Pemphigus vulgaris

#### 4.1.1 Cytokine network in pemphigus vulgaris

**Figure 2:**
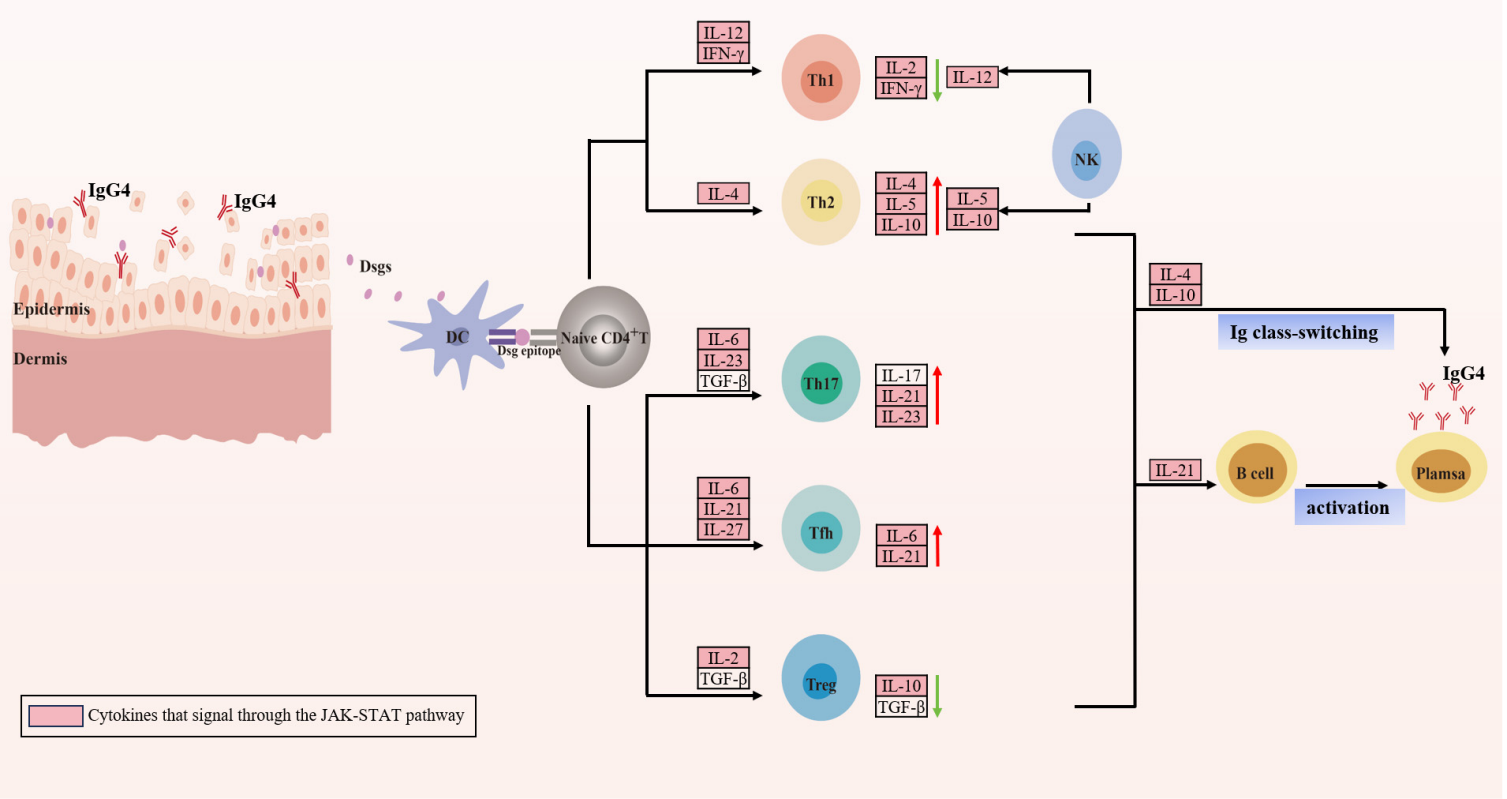
Cytokine involvement in pemphigus vulgaris. Exposed desmogleins (Dsgs) identified by antigen-presenting cells such as dendritic cells and present the antigen to autoreactive naïve CD4^+^T cells. Subsequently, under the influence of various cytokines, these T cells differentiate into distinct subsets, secreting cytokines that predominantly exert their biological effects via the JAK-STAT pathway. Specifically, Th2 differentiation is enhanced, leading to the secretion of Th2 cytokines IL-4, IL-5, and IL-10, while simultaneously suppressing Th1 responses and downregulating Th1 cytokines such as IL-2 and IFN-γ. This shift may be associated with cytokines released by NK cells. Additionally, Th17 and Tfh cell differentiation is amplified, resulting in the secretion of IL-6, IL-17, IL-21, and IL-23. Notably, IL-6 and IL-17 promote inflammatory responses, IL-21 enhances B cell activation into plasma cells, and IL-4 and IL-10 facilitate antibody class switching, prompting plasma cells to secrete IgG4 antibodies against Dsgs, culminating in acantholysis. Furthermore, a reduction in Treg cell differentiation is crucial for the proliferation of autoreactive T cells and antibody production. Figure image created with Adobe Illustrator2023.

PV is a severe organ-specific AIBD. The underlying cause is the production of autoantibodies that attack the essential proteins Dsg1 and/or Dsg3. This attack results in the loss of cell adhesion of keratinocytes, a process known as acantholysis. This process ultimately leads to the formation of blisters and erosion of the skin and mucous membranes (4). Multiple factors, including drugs, environment, infections, and others, are known to initiate the formation of autoantibodies that expose Dsgs. Once exposed, these Dsgs are recognized by APCs such as DCs, macrophages, or B cells, which present them to T cells through the MHC II-antigen complex interaction. In susceptible individuals, T cells with autoreactive potential have evaded both central and peripheral clonal deletion. Upon recognition of the MHC II-self peptide ligand complex and in the presence of inflammatory mediators, these T cells are activated and differentiate into Th1, Th2, Th17, Tfh, or Treg. Ultimately, these cells secrete various cytokines that can drive B-cell proliferation, activation, pathogenic DSG-specific IgG antibody production, as well as differentiation into plasma cells and memory cells (**Figure 2**) (167).

##### 4.1.1.1 Th1 and Th2 cytokines in PV

Th1 cells are primarily responsible for secreting IFN-γ and IL-2, which play a crucial role in cellular immunity against intracellular microorganisms. In contrast, Th2 cells secrete cytokines such as IL-4, IL-5, IL-10, and IL-13, which are associated with host immunity to extracellular pathogens and the development of allergic diseases (168). Early studies suggest that PV pathogenesis involves both Th1 and Th2 cells. During the active phase of PV, DSG3-reactive Th2 cells secreting IL-4 are predominant. Conversely, during the chronic phase, DSG3-reactive Th1 cells secreting IFN-γ become more prevalent (169). This pattern correlates with the production of IgG4 antibodies during active disease and IgG1 antibodies during clinical remission. Additionally, there is a significant correlation between the presence of IgG1 and IgG4 antibodies against Dsg3 and the ratio of Dsg3-reactive Th1/Th2 cells (170). However, recent evidence has highlighted the critical role of Th2 cells in the development of PV. By constructing mouse or human Dsg-3 reactive T cell lines, researchers have found that Dsg-3 reactive T cells predominantly secrete Th2 cytokines IL-4 and IL-10 both *in vivo* and *in vitro* (171, 172). Further studies on PV patients have shown that the number of DSG-reactive Th2 cells is positively correlated with Dsg-3 antibody titer and disease activity (173). Moreover, the expression of Th2 cytokines such as IL-4, IL-5, and IL-10 was significantly increased during the active phase of the disease but decreased after disease control (174, 175). Dupilumab, a fully human monoclonal antibody that simultaneously blocks IL-4 and IL-13 signals by targeting IL-4Rα, is anticipated to be a valuable addition to the therapeutic intervention for PV (176), as demonstrated in several case reports in recent years (177–179).

Upon differentiation of the naïve T cells into Th2, they release Th2 cytokines that impede the Th1 response and down-regulate the production of Th1 cytokines (180). This phenomenon has been substantiated in patients with PV. The study conducted by Satyam et al. has verified that PV patients exhibited elevated levels of serum Th2 cytokines (IL-4 and IL-10) and reduced levels of Th1 hallmark cytokines (IFN-γ and IL-2) (174). Analogously, other studies have also affirmed that patients with PV exhibit reduced levels of serum IFN-γ and heightened levels of IL-4. This suggests that during the active phase of the disease, the Th2 response inhibits the Th1 response (181, 182). The underlying reason might be attributed to the functional alterations in NK cells in PV patients. Studies have demonstrated that the peripheral blood of PV patients contains an increased number of NK cells, while the IL-12 signaling pathway is impaired, and the expression of IL-5 and IL-10 is elevated in NK cells, thereby promoting a predisposition toward a Th2 type immune response in PV patients (183).

##### 4.1.1.2 Th17 cytokines in PV

Upon exposure to cytokines TGF-β, IL-23, and IL-6, T cells undergo differentiation into Th17 cells, characterized by the secretion of a range of cytokines including IL-17, IL-21, IL-22, IL-26, and GM-CSF. Several studies have reported a significant increase in the number of Th17 cells in the peripheral blood and skin lesions of PV patients compared to the general population (184–186). Yang et al. (187) found that in PV skin lesions, the predominant T cell population was composed of CD4^+^ T cells expressing IL-21 and IL-17A, rather than classical Tfh cells expressing CXCR5. They speculate that these IL-21+/IL-17+ CD4^+^ T cells may contribute to the activation and differentiation of B cells, ultimately leading to the production of pathogenic autoantibodies in PV lesions. Additionally, another study discovered a greater number of CD154+ CD4^+^ T cells in the peripheral blood of PV patients, which highly expressed IL-17 and IL-21 and positively correlated with the level of Dsg-3 titer (188). Analyses for Th17-related cytokines revealed significantly elevated serum levels of IL-17, IL-21, and IL-23, but decreased levels of IL-22 in patients with PV compared to normal controls (189–191), and similar results were obtained in PV lesions (186, 192). Andrés et al. (193) reported the treatment of a PV patient with ustekinumab, an inhibitor of the p40 subunit of IL-12 and IL-23, after one month, the patient showed significant improvement in clinical symptoms and reduced serum levels of IL-12, IL-17, IFN-γ, and IL-6 without any adverse effects. However, as the treatment continued, a complete relapse of clinical symptoms was observed.

##### 4.1.1.3 Tfh cytokines in PV

Tfh cells are a specialized subset of CD4^+^ T cells that localize to lymphoid follicles. Under the influence of cytokines such as IL-6, IL-21, and IL-27, naïve T cells induce the expression of Bcl-6 through the activation of transcription factors STAT3 or STAT4, thereby promoting differentiation along the Tfh lineage. Tfh cells can secrete cytokines like IL-21, IL-6, and IL-10, which support the survival and proliferation of B cells in the germinal center. This process ultimately drives B cell differentiation into plasma cells, immunoglobulin class-switching, and affinity maturation of antibodies (194). Tina et al. (195) found that IL-27 levels in the plasma of PV patients were positively correlated with Dsg-specific autoantibodies, as well as a significant increase in the frequency of Tfh cells and IL-21 levels, a cytokine produced by both Th17 and Tfh cells. Two other recent studies have also confirmed that Tfh cell frequencies are increased in PV patients and are strongly correlated with Dsg antibody levels, and that serum levels of cytokines such as, IL-6, and IL-21 are also significantly elevated (196, 197). However, there have been no reports on the function of IL-21 and the effects of anti-IL-21 therapy in PV.

##### 4.1.1.4 Treg cytokines in PV

Previous studies have detected Dsg3-specific T cells in healthy individuals carrying PV-associated HLA class II alleles (198, 199), However, these genetically susceptible individuals do not manifest PV phenotypes, which is attributed to the presence of IL-10- and TGF-β-secreting Dsg-3-specific Treg cells in this population—a cell type rarely found in PV patients (200). Naive T cells, upon stimulation by IL-2 and TGF-β, differentiate into Treg cells, which predominantly secrete TGF-β and IL-10 for immunosuppressive and immunomodulatory functions (201). Several studies have indicated that the proportion of Treg cells in the peripheral blood of PV patients is significantly reduced (202, 203) and negatively correlates with the number of Th17 cells (185, 191), which the latter is significantly increased in PV patients. Increasing Treg cell numbers through adoptive transfer or using the superagonistic anti-CD28 antibody D665 inhibits the production of Dsg-3 autoantibodies in a mouse model of PV, while Treg depletion enhances autoantibody production (204). A recent study found that Dsg3-specific CD4^+^ T cells could overcome peripheral tolerance in regulatory T cell depleted mice, inducing skin lesions, but not in wild-type mice (205). However, cytokine-level studies have found elevated levels of TGF-β and IL-10 in PV patients compared to normal subjects, suggesting the existence of other cellular sources of secretion besides Treg cells (174, 191, 206, 207). An explanation for this may relate to the pleiotropic nature of these cytokines; for instance, TGF-β contributes to the differentiation of Tregs while also promoting the development of Th17 cells. Similarly, IL-10 supports the differentiation of Tregs and regulatory B cells and acts as a key member of the Th2 cytokine family. Notably, PV patients have shown significant increases in both Th17 and Th2 responses.

##### 4.1.1.5 Other proinflammatory cytokines in PV

IL-6, IL-8, TNF-α, and IL-1 are archetypal pro-inflammatory cytokines predominantly secreted by non-Th immune cells. Elevated levels of these cytokines have been observed in PV patients and are known to decrease post-treatment, aligning with disease activity (175, 206, 208–215). TNF-α and IL-1 are implicated in promoting keratinocyte acantholysis via C3 complement activation, plasminogen activator induction, or through independent mechanisms (211, 216). Notably, PV-susceptible mice lacking TNF-α and IL-1 receptors show attenuated disease upon passive transfer, underscoring the role of these cytokines (211). Emerging research highlights TNF-α’s capacity to compromise keratinocyte adhesion by upregulating ST18 in PV lesions (217). Encouragingly, TNF-α inhibitors, such as etanercept and infliximab, have been instrumental in managing refractory PV cases (218–221). Additionally, IL-6 receptor monoclonal antibodies have demonstrated therapeutic success in PV (222). However, the therapeutic potential of IL-8 and IL-1 monoclonal antibodies remains unexplored in PV.

In aggregate, cytokines including Th2 (IL-4, IL-5, IL-10), Th17 (IL-17, IL-21, IL-23, TGF-β), and other pro-inflammatory cytokines (IL-6, IL-8, TNF-α, IL-1) exhibit heightened expression in PV, with certain cytokines correlating positively with disease severity and autoantibody titers, and their diminished expression post-successful treatment. These cytokines have a significant role in promoting T-cell differentiation, B-cell maturation, and antibody production, indicating their crucial involvement in the pathogenesis of PV, rather than transcending a mere epiphenomenon during disease evolution. Despite the pronounced role of these cytokines, monoclonal antibodies targeting individual cytokines have not matched the anticipated therapeutic impact observed in conditions like psoriasis (223), AD (224), or RA (225). This divergence suggests a multi-cytokinetic interplay in PV, advocating for therapeutics targeting a constellation of cytokines. Notably, cytokines including IL-4, IL-5, IL-6, IL-10, IL-17, IL-21, and IL-23 exert their effects via the JAK-STAT pathway, with IL-8, TNF-α, IL-1, and TGF-β’s expression being modulated indirectly by this pathway. Consequently, JAK inhibitors emerge as a promising therapeutic contender for PV.

#### 4.1.2 JAK inhibitors in PV

A research group scrutinized the expression levels of JAK3, STAT2, STAT4, and STAT6 proteins in skin and oral mucosal lesions of PV patients through immunohistochemistry. Their findings indicated a significant upregulation of these proteins in the lesions compared to those in healthy controls. The authors hypothesize that this upregulation correlates with the heightened levels of various cytokines in PV patients and propose that JAK/STAT proteins could emerge as novel therapeutic targets for PV (226, 227). Given that the activation of the JAK-STAT pathway is contingent upon the phosphorylation status of JAK and STAT proteins, and considering that cytokines implicated in PV primarily operate through STAT3 and STAT6, we assessed the levels of phosphorylated STAT3 and STAT6 in the skin lesions of PV patients and healthy controls. The results showed that pSTAT3 and pSTAT6 expression were significantly elevated in PV lesions (unpublished data).

Although clinical trials on JAK inhibitors for PV treatment are lacking, Tavakolpour et al. (228). regard tofacitinib, a JAK1/3-targeting small molecule inhibitor, as a potential therapeutic agent for refractory PV. Tofacitinib has been reported to inhibit a spectrum of cytokines from diverse cellular origins across a range of diseases, including IL-4, IL-5, IL-6, IL-10, IL-17, IL-21, IL-23, IL-8, TNF-α, and IL-1 (229–232), all of which are intricately linked to PV pathogenesis. Vander et al. (233). presented a case of PV with severe nail involvement, where a combination treatment of tofacitinib and rituximab led to a significant amelioration of skin and nail conditions within three weeks. The authors attribute the swift symptom improvement to tofacitinib, given rituximab’s protracted therapeutic onset. M Grace et al. recently reported the successful treatment of refractory oral mucosal lesions in a patient with PV using upadacitinib (234). Our team recently administered a treatment regimen of tofacitinib 5mg twice daily, in conjunction with prednisone 1mg/kg daily, to a patient with refractory PV. This treatment led to a noticeable improvement in the patient’s skin lesions and a decrease in corticosteroid reliance (unpublished data). These outcomes hint at tofacitinib’s potential as an efficacious drug for PV management. Nonetheless, further clinical trials are imperative to substantiate these findings. To date, no clinical applications of other JAK inhibitors in PV have been documented (**Table 2**).

**Table 2:** Targeting characteristics of JAK inhibitors, rituximab, dupilumab and their applicability in PV/BP.

JAK Inhibitor	Targeted JAK Subtypes	Major PV/BP-Related Pathways Inhibited	Potential Advantages	Limitations	Applicable Diseases	Current Evidence Level
Tofacitinib	JAK1/3	IL-4, IL-6, IL-17, IL-23, IFN-γ	Broad inhibition of Th1/Th2/Th17 pathways; Rapid onset (2-3 weeks); Synergistic effect with rituximab	High infection risk; Off-target JAK2 inhibition may cause anemia; Cardiovascular event risk	PV, BP	PV: 5 case reports; BP: 10 case reports
Baricitinib	JAK1/2	IL-5, IL-12, IL-23, IFN-γ	Strong inhibition of Th1/Th17 axis; Effective against eosinophil activation (JAK2-dependent); Once-daily dosing convenience	JAK2 inhibition increases anemia risk; Hepatotoxicity risk; Lack of PV data	BP	BP: 1 case report
Upadacitinib	JAK1	IL-4, IL-13, IL-23	High selectivity for JAK1 (reduced off-target effects); Strong mucosal penetration (suitable for oral PV); Lower infection risk compared to tofacitinib	Weak inhibition of Th1 pathway; Optimal dosing for BP not established; Higher cost	PV, BP	PV: 3 case reports; BP: 3 case reports
Abrocitinib	JAK1	IL-4, IL-13, IL-31 (Th2 pathway)	Strong reduction in IgE levels Significant itch relief Excellent skin penetration	Ineffective against Th17 pathway; Only suitable for IgE-mediated BP subtype; Lack of long-term safety data	BP	BP: 3 case reports
Rituximab	Anti-CD20 monoclonal antibody	B cell depletion	High efficacy in PV	Delayed onset (8-12 weeks)	PV	PV: RCTs
			Long-term disease control	Infusion reactions		
Dupilumab	IL-4Rα blocker	IL-4/IL-13 (Th2 pathway)	Rapid onset (4-6 weeks)	Limited efficacy in Th1/Th17-driven disease	BP	BP: Case series
			Safe profile (low infection risk)	Injection-site reactions		
			Effective in Th2-dominant BP			

### 4.2 Bullous pemphigoid

#### 4.2.1 Cytokine network in bullous pemphigoid

**Figure 3:**
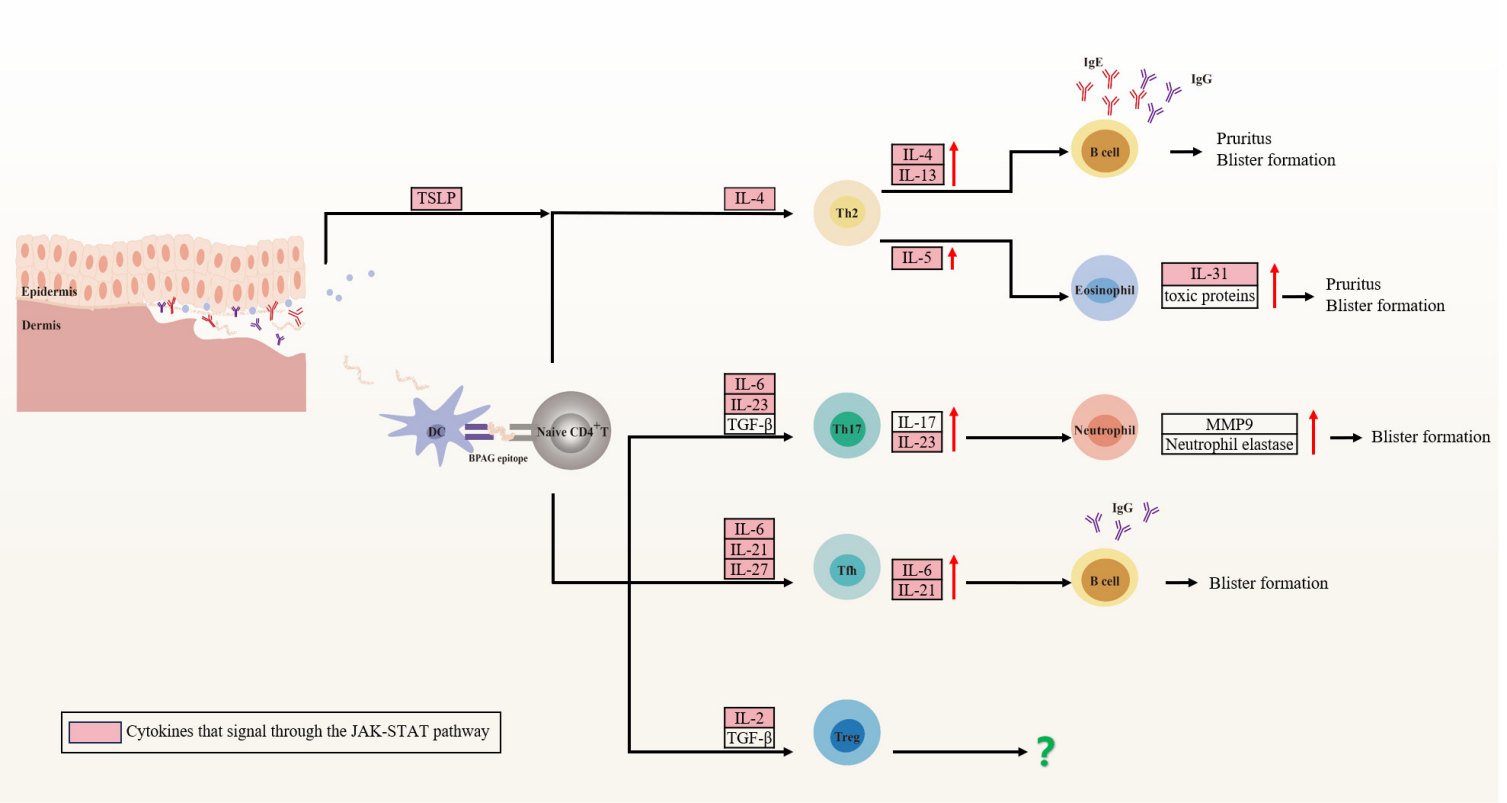
Cytokine involvement in bullous pemphigoid (BP). Patient-specific factors and external stimuli lead to the exposure of BPAG antigens, recognized and presented by dendritic cells to naïve CD4+ T cells. These cells differentiate into CD4+ T cell subsets that secrete cytokines inducing pruritus and vesicle formation, predominantly through the JAK-STAT pathway. Specifically, Th2 cells secrete IL-4 and IL-13, which regulates IgG and IgE isotype switching, while IL-5 enhances eosinophil accumulation and activation. Eosinophils secrete IL-31 and toxic proteins, contributing to local inflammation. Th17 cells produce IL-17 and IL-23, activating neutrophils that release neutrophil elastase and MMP-9, degrading the extracellular matrix and disrupting dermal-epidermal junctions. Tfh cells stimulate high-affinity autoantibody production by B cells via IL-21. However, the role of Treg cells in BP remains controversial. Figure image created with Adobe Illustrator2023.

BP, akin to pemphigus, originates from a multifactorial interplay among patient-specific elements such as genetic susceptibility, regulatory Treg dysregulation, and aging, as well as external inciters like physical trauma, infections, malignancies, and medications. These elements may synergistically precipitate the collapse of immune tolerance to the BPAG protein, culminating in autoantibody production and disease manifestation (235). Specifically, DCs present a BPAG epitope via MHC class II, which subsequently activate BPAG-specific CD4^+^ T cells. These activated T cells further differentiate into distinct Th cell subsets, releasing a spectrum of cytokines encompassing IL-4, IL-13, IL-5, IL-6, IL-17, IL-21, and IL-31. This cytokine milieu fosters B cells differentiation and the production of IgG and IgE autoantibodies (236). The autoantibodies then bind to the basement membrane zone of the skin, instigating subepidermal blistering in BP via both complement-dependent and complement-independent mechanisms (**Figure 3**) (237, 238).

##### 4.2.1.1 Role of type 2 inflammation-related cytokines in BP

In contrast to PV, BP is characterized by not only blister formation but also by the frequent presence of erythema, urticaria, eczematous-like rashes, and intense pruritus. This is due to the involvement of IgE and various innate immune cells, such as eosinophils and mast cells, in the pathogenesis of BP, indicating a close association between BP and type 2 inflammation. Indeed, Multiple studies have demonstrated elevated levels of type 2 inflammation-related cytokines, including IL-4, IL-5, IL-13, IL-31, and TSPL in BP patients (239–242).

IL-4 is particularly implicated in BP pathogenesis due to its role in Th2 cell differentiation and its facilitation of B cell immunoglobulin class switching to IgG1 and IgE (56). Pickford et al. reported that peripheral blood mononuclear cells from BP patients exhibit a significant increase in IL-4 secretion upon exposure to NC16A peptides (243). Furthermore, recent research has identified two major epitope peptides within BP180-NC16A that correlate with the induction of IL-4 production and autoantibody secretion in BP (244). IL-13, which shares functional similarities with IL-4 through its action on the IL-4Rα, is also implicated in BP. It synergizes with IL-4 to enhance B cell differentiation and IgE production (224). Researchers have reported the association between IL-13 gene polymorphism and the risk of BP (245, 246). In addition, IL-13 levels have been found to be positively correlated with the severity of pruritus in BP (247).

Due to their pivotal role in the pathogenesis of BP, several targeted therapeutic agents have been developed and are now in clinical use. Among these, dupilumab has garnered considerable attention. Since Alex et al. first reported the successful treatment of a refractory case of BP with dupilumab in 2018 (248), multiple case reports, case series, retrospective cohort studies, and systematic reviews have further substantiated its efficacy (249–252). These studies consistently demonstrate that dupilumab is a safe and effective therapeutic option, and its combined use with corticosteroids or immunosuppressants is recommended for the treatment of moderate to severe BP (253). Furthermore, a multicenter, randomized, double-blind, placebo-controlled clinical trial (NCT04206553) is underway, led by Dédée et al., to assess the efficacy and safety of dupilumab in adult patients with BP (254). Recently published data demonstrate that the dupilumab treatment group achieved all primary and secondary endpoints, including a significantly higher proportion of patients achieving complete remission, reduced disease severity, and alleviated pruritus compared to the control group, with no differential safety profile observed (255). Tralokinumab, a monoclonal antibody targeting IL-13, has also been documented in the successful treatment of a patient with BP complicated by end-stage kidney disease (256).

IL-5 serves as a pivotal cytokine for the maturation, survival, and functional activity of eosinophils, with its levels correlating with the severity of BP (257). In the presence of BP autoantibodies, IL-5 can activate eosinophils, which are implicated in the direct promotion of dermal-epidermal separation (258). Furthermore, another study has demonstrated that IL-5 is elevated in BP blister fluid, and is essential for the release of toxic proteins by eosinophils and the detachment of keratinocytes (259). Immunotherapies targeting the neutralization of IL-5 and its receptor have been documented in the treatment of, including mepolizumab, reslizumab, and benralizumab (260). A phase 2 double- blind trial (NCT01705795) of mepolizumab in BP demonstrated a significant reduction in peripheral blood eosinophil counts among treated patients. However, there were no significant differences in the proportion of patients remaining relapse-free or in the median time to relapse between the mepolizumab and placebo groups (261). For reslizumab, to date, two cases of refractory BP have been reported successfully managed with reslizumab, highlighting its efficacy in treating this condition (262, 263). In addition, recently, Arisa et al. reported a severe asthma patient developing BP during treatment with benralizumab. The authors hypothesized that BP may paradoxically arise through diverse mechanisms, even with therapeutic agents typically efficacious against the condition (264). A phase 3, double-blind clinical trial (NCT04612790) is currently evaluating benralizumab for BP treatment.

IL-31 serves as a principal inducer of pruritus and predominantly produced by eosinophils, is posited to exert immunomodulatory effects, specifically by promoting Th2-type immune responses (265). Elevated levels of IL-31 were detected in both lesional tissue and serum in BP patients (266, 267), and chemotactic and stimulated eosinophils to release reactive oxygen species and the chemokine CCL26 (268). While IL-31’s contribution to BP-associated itching is recognized, its role as the principal mediator of pruritus in BP remains to be definitively established. Nemolizumab, a monoclonal antibody directed against the IL-31RA, has shown efficacy in reducing pruritus in AD and prurigo nodularis (269). This suggests that nemolizumab may also be effective in the treatment of pruritus in BP, underscoring the need for further exploration of its therapeutic potential in this condition.

TSLP is a critical initiator of type 2 allergic responses, activating DCs, ILC2, naïve CD4^+^ T cells, and Th2 cells, and is implicated in the pathogenesis of itching (270). Elevated concentrations of TSLP have been reported in skin lesions, blister fluid, and sera of patients with BP (271–274). Dysfunction of BP180 has been shown to upregulate TSLP expression in keratinocytes, and this upregulation is strongly correlated with the severity of itch in a mouse strain. Moreover, the administration of an anti-TSLP neutralizing antibody could result in diminished scratching behavior, indicating the potential therapeutic efficacy of TSLP inhibition (271). Although no clinical trials have yet been conducted on TSLP inhibitors for BP treatment, Tezepelumab—a human monoclonal antibody targeting TSLP—is currently in trials for asthma and AD (275). Given its potential, Tezepelumab may emerge as an adjunctive therapy in BP treatment, offering a novel approach to alleviate pruritus and enhance the quality of life for patients.

##### 4.2.1.2 Role of Th17 cytokines in BP

The role of IL-17 and IL-23 in the pathogenesis of BP has been underscored by multiple studies demonstrating elevated levels of these cytokines in lesional skin, blister fluid, and serum of BP patients (276–280). Notably, persistently increased serum levels of IL-17 and IL-23 have been correlated with a higher risk of disease relapse post-treatment initiation (280). Genetic associations have also been identified, with two single-nucleotide polymorphisms (rs2201841 and rs7530511) in the IL-23R linked to BP (281). The absence of the NC14A domain of BP180 in mice triggers an autoimmune response against the cutaneous basement membrane, which is mitigated by anti-IL-17A treatment (282). IL17A-deficient mice exhibit resistance to autoantibody-induced BP, and pharmacological inhibition of IL-17A has been shown to reduce BP induction (283). These cytokines are implicated in BP development through the upregulation of MMP-9 and neutrophil elastase expression, processes that facilitate the separation of the dermis and epidermis (284, 285). Additionally, IL-17 and IL-23 have been shown to upregulate the expression of the glucocorticoid receptor-β, potentially contributing to glucocorticoid resistance in BP (286). Collectively, these findings suggest that the IL-17 axis plays a functional role in BP, and targeting this pathway with biologics presents a promising therapeutic strategy.

Indeed, targeting the IL-17/IL-23 axis has shown efficacy in patients with coexisting BP and psoriasis (287, 288). However, there are reports of new-onset BP in individuals treated with ustekinumab, secukinumab, or guselkumab for psoriasis, highlighting the complexity of cytokine-targeted therapies (289–291). A phase 2 open-label clinical trial (NCT03099538) investigating ixekizumab, a monoclonal antibody against IL-17A, did not meet its primary endpoint, as no cessation of blister formation within 12 weeks. Despite this setback, ongoing clinical trials are exploring the efficacy of biologics targeting IL-12/23 (Ustekinumab, NCT04117932) and IL-23 (Tildrakizumab, NCT04465292) in BP patients.

##### 4.2.1.3 Tfh and IL-21 in BP

Tfh cells play a pivotal role in the generation of high-affinity autoantibodies by B cells within germinal centers through the secretion of IL-21 (292). The proportion of circulating Tfh cells and the plasma levels of IL-21 have been found to be markedly elevated and exhibit a positive correlation with both anti-BP180-NC16A autoantibody titers and disease severity in BP. *In vitro* studies have shown that the depletion of Tfh cells or the blockade of IL-21 can effectively suppress T cell-mediated B cell activation and the secretion of BP autoantibodies (293). Ohuchi et al. have reported an increase in both CXCL13, a chemokine critical for Tfh cell homing to germinal centers, and Tfh cells in the lesional skin and peripheral blood of BP patients, with a positive correlation to serum anti-BP180-NC16A titers (294). Furthermore, in STAT6-deficient scurfy mice, which are incapable of Tfh cell development, the Tfh cell population is markedly reduced, and these mice exhibit diminished production of autoantibodies against BP antigens (295). Collectively, these findings underscore the importance of the Tfh/IL-21 axis in the immunopathogenesis of BP and suggest that modulation of this pathway could represent a promising therapeutic approach for the disease.

##### 4.2.1.4 Treg and IL-10 in BP

The presence of Tregs and the cytokine IL-10 in peripheral blood and skin of BP patients is a subject of ongoing debate. Some studies have reported a decrease in the frequency of Tregs and IL-10 levels in BP, correlating with disease activity, while others have yielded conflicting results (240, 296–299). Nonetheless, it is established that Tregs are crucial for maintaining peripheral immune tolerance by suppressing the activation and proliferation of autoreactive T cells. In both human conditions such as immune dysregulation, polyendocrinopathy, enteropathy, X-linked (IPEX) syndrome, and in animal models like scurfy mice, Treg deficiency is associated with the spontaneous generation of autoantibodies against BP180 and BP230, suggests a significant role for Tregs in the regulation of autoantibody production in BP (295, 300). However, the precise role of Tregs in the pathogenesis of BP—whether they play a primary or ancillary role—remains to be elucidated through further investigation.

##### 4.2.1.5 Other proinflammatory cytokines in BP

Elevated expression of multiple pro-inflammatory cytokines, including IL-1β, IL-6, and TNF-α, has been observed in the serum of patients with BP and correlates positively with disease severity (207). IL-6 may be implicated in the development of BP by promoting the differentiation of Th17 and Tfh cells, as well as the production of antibodies by B cells (301). TNF-α can induce the release of inflammatory mediators such as IL-1, IL-6, IL-8, eotaxin-1, and MMP-9, and modulate the differentiation of T and B cells (302). Similarly, IL-1β can facilitate the release of TNF-α, IL-6, and MMP-9, leading to the recruitment of immune cells and the development of T-cell and B-cell-driven inflammatory responses (303). Despite the association of these cytokines with BP pathogenesis, their specificity in the disease is constrained. To date, no studies targeting IL-6/IL-6R or IL-1β for the treatment of BP have been reported. The application of TNF-α inhibitors in BP treatment has been documented in several case reports with inconsistent outcomes (304, 305), and in some instances, the use of TNF-α inhibitors has been associated with the onset of BP (306).

The presented data indicate that, similar to pemphigus vulgaris, the pathogenesis of BP is primarily linked to Th2 (IL-4, IL-5, IL-13, IL-31) and Th17 (IL-17, IL-23) cytokines. These cytokines are variably involved in the production of autoantibodies, the recruitment of eosinophils and neutrophils, the induction of pruritus, and the formation of bullae. Clinical trials targeting individual cytokines with biologic agents are currently in progress, with some showing favorable therapeutic outcomes, while others have demonstrated less satisfactory results. This variability suggests that BP is not a disease dominated by a single cytokine. Therefore, the concurrent inhibition of multiple cytokines, such as with JAK inhibitors, may offer a novel and potentially efficacious therapeutic alternative for this condition.

#### 4.2.2 JAK inhibitors in bullous pemphigoid

The involvement of the JAK/STAT signaling pathway in the pathogenesis of BP has been established. Juczynska et al. reported increased expression of all STAT proteins and JAK2 and JAK3 in BP skin lesions, and they proposed that JAK2 is implicated in the signaling pathways of IFN-γ and IL-5, whereas JAK3 predominantly influences the IL-4 and Th17 axes (307). Our recent findings indicate significantly elevated levels of phosphorylated STAT3 and STAT6 in BP lesions, reflecting overactivation of the JAK-STAT pathway at both the protein and transcriptional levels, as confirmed by immunohistochemistry (IHC) and transcriptome sequencing. Notably, these levels were substantially reduced following treatment with the JAK inhibitor tofacitinib (308). A review of the literature identified 17 cases of BP patients treated with JAK inhibitors since 2022 (309–317). The JAK inhibitors used included tofacitinib (JAK1/3, in 10 patients), baricitinib (JAK1/2, in 1 patient), upadacitinib (JAK1, in 3 patients), and abrocitinib (JAK1, in 3 patients). The treatment showed high efficacy and an acceptable safety profile (**Table 2**).

In summary, while JAK inhibitors present promising new therapeutic avenues for managing refractory PV/BP, the absence of large-scale randomized controlled trials (RCTs) and head-to-head comparison of JAK inhibitors and current first-line treatments— such as rituximab, corticosteroids, and dupilumab—limits our understanding of their relative efficacy, safety, and cost-effectiveness in PV/BP patients.

## 5 Safety issues of JAK inhibitors

The safety profile of JAK inhibitors was initially regarded as comparable to biologic DMARDs (bDMARDs) based on early clinical trials in rheumatoid arthritis (RA) patients without severe comorbidities, which showed no significant increase in major adverse cardiovascular events (MACEs), venous thromboembolism (VTE), or malignancies, except for a higher risk of herpes zoster (318–327). However, this perspective shifted after the FDA’s 2021 black box warning, prompted by findings from the ORAL Surveillance trial (A3921133; NCT02092467).

The ORAL Surveillance was a 4-year, randomized, open-label, non-inferiority, post-authorization safety endpoint trial, compared tofacitinib (5 mg or 10 mg twice daily) with TNF inhibitors (etanercept or adalimumab) in RA patients (aged ≥50 years with at least one additional cardiovascular risk factors).The risks of MACE and cancers (excluding nonmelanoma skin cancer) were higher with the combined tofacitinib doses compared to TNF inhibitors, and the non-inferiority of tofacitinib was not demonstrated (328). Specifically, for tofacitinib at the approved dose of 5 mg twice daily, the ORAL Surveillance trial showed a numerically but not statistically higher risk of MACEs, serious infections, adjudicated hepatic events, VTE, deep vein thrombosis, pulmonary embolism, or adjudicated death from any cause compared to a TNF inhibitor. Additionally, there was a numerically and statistically significant increase in the risk of malignancies and adjudicated opportunistic infections, including herpes zoster and tuberculosis, as well as all cases of herpes zoster (both nonserious and serious) (328).

Following these results, FDA expanded safety warnings to all JAK inhibitors (baricitinib, upadacitinib, filgotinib), and the European Medicines Agency emphasizing caution in high-risk populations, including patients aged ≥65 years, smokers, and those with cardiovascular/metabolic comorbidities or prior histories of malignancies or VTE (329, 330). Subsequent *post-hoc* analyses of the ORAL Surveillance trial data have further elucidated which patients are at the highest risk for MACEs, malignancies, VTE, and death: (1) Patients with high risk or history of ASCVD have the highest risk of MACEs, and the appropriate use of statin therapy should be considered (331). (2) The increased incidence of malignancies is associated with a history of MACEs or ASCVD (332). (3) The risk of VTE is heightened in patients with a prior history of VTE, active RA, advanced age, obesity, or those undergoing hormone replacement therapy, when treated with either tofacitinib or TNF inhibitors. The causal role of JAK inhibitors in VTE remains inconclusive (333). (4) Elderly patients, smokers, and those with active disease are more susceptible to infections when treated with JAK inhibitors (334, 335). (5) Events including MACEs, malignancies, VTE, and serious infections nearly exclusively occur in “high-risk” patients (aged ≥ 65 years and/or former smokers), and are seldom seen in “low-risk” patients (aged < 65 years with no smoking history) (336). These studies provide valuable insights into better understanding of the true risks associated with JAK inhibitor use in clinical practice and how to mitigate such risks.

However, the ORAL Surveillance study has also raised several unresolved issues. Firstly, sustained systemic inflammation and disease activity have been identified as significant contributors to the development of MACEs, malignancies, VTE, and infections in patients with RA (337, 338). It remains contentious whether the adverse events observed in the ORAL Surveillance study are due to the disease itself or to the toxicity of tofacitinib. As several studies utilizing surrogate markers have revealed that JAK inhibitors can mitigate cardiovascular risk by reducing inflammation (339, 340). Secondly, the ORAL Surveillance study lacks a comparator group treated with placebo or conventional synthetic DMARDs (csDMARDs). This limitation precludes answering whether JAK inhibitors reduce the incidence of MACEs, malignancies, and VTE compared to inadequate treatment or csDMARD therapy in RA patients. The study only indicates that JAK inhibitors may not be as effective as TNF inhibitors in reducing the risk of these events, as TNF inhibitors have been shown to decrease the occurrence of MACEs (341).

Several clinical trials, meta-analyses, and integrated safety analyses for dermatological conditions such as AD, psoriasis, and AA have also confirmed the favorable safety profile of JAK inhibitors (342–344). Common adverse reactions include respiratory tract infections, nasopharyngitis, headache, and herpes zoster, all of which are self-limiting or can be alleviated with symptomatic treatment. The incidence of serious adverse events, such as MACEs, malignancies, and VTE, is not different from that of the control group. Similar to the ORAL Surveillance study, the incidence of these serious adverse events is also higher in dermatological patients aged 65 or older or those with a history of smoking (345). Due to the limited application of JAK inhibitors in AIBD, there have been no reports of serious adverse events to date. However, since pemphigus and pemphigoid diseases predominantly affect the elderly, and these patients have a higher risk of malignancies (346), VTE (347), and MACEs (348) compared to the general population, vigilance is warranted when using JAK inhibitors. Accordingly, prior to treatment initiation, a detailed medical history should be obtained to assess for VTE, malignancies, and associated risk factors. A comprehensive blood count, liver and kidney function tests, lipid panel, and screenings for hepatitis B, hepatitis C, HIV, and tuberculosis are recommended. Adult patients are advised to receive pneumococcal and herpes zoster vaccinations. Routine monitoring during treatment is essential, with particular attention to complete blood count, creatinine clearance, kidney and hepatic function. Dose adjustment or discontinuation is recommended in cases of significant hemoglobin decline (greater than 2 g/dL or levels below 8 g/dL), absolute neutrophil count between 500–1000/mm³, absolute lymphocyte count between 500–750/mm³, creatinine clearance between 30–60 mL/min or less than 30 mL/min, or in the presence of severe hepatic impairment (349).

## 6 The selectively of JAK inhibitors

JAKs are composed of approximately 1000 amino acids, with molecular weights ranging from 120 to 140 kDa, and consist of seven homology domains (JH1-7). The JH1 domain is the active kinase catalytic domain, containing conserved tyrosine residues that are the principal targets for JAK inhibitors. The high degree of homology among the JH1 domains of different JAK isoforms presents a challenge for the development of highly selective inhibitors. Selectivity of JAK inhibitors is evaluated *in vitro* assays using purified enzymes or cytokine stimulation with assessment of pSTAT activation. *In vitro* kinase assays and cellular assays have demonstrated that tofacitinib selectively inhibits cytokines signaling through JAK1 and JAK3 over JAK2, baricitinib shows specificity for JAK1 and JAK2 over JAK3, and both upadacitinib and abrocitinib are identified as selective JAK1 inhibitors (350–352). **Table 3** illustrates the enzymatic and whole-cell activities of JAK inhibitors reported for the treatment of BP, where a lower IC50 value indicates greater potency. Selectivity for a specific JAK isoform is determined by the IC50 values and the ratios between them for the different JAK isoforms. The outcomes are contingent upon the assay substrates, cell lines, and cytokines being measured.

**Table 3:** The enzymatic and whole-cell activities of JAK inhibitors reported for the treatment of BP.

Compound	Enzyme assay IC50 (nM)*				Human whole blood IC50 (nM)				
	JAK1	JAK2	JAK3	TYK2	IL‐15^‡^ pSTAT5	IL-6^§^ pSTAT1	IL-12^||^ pSTAT4	IFNα^¶^ pSTAT3	IL-23^||^ pSTAT3
Tofacitinib	15.1	77.4	55	489	55.8	75.4	409	35	229
Baricitinib	4	6.6	787	61	259	21.1	149	28.7	81.9
Upadacitinib	47	120	2304	4690	182	66		90	1683
Abrocitinib	29.2	804	>10000	1250	1298(CD8+)	16.3(CD14+)	13673	18.9	>16452

*All compounds were assayed at 1 mM ATP

‡IL‐15 signals through JAK1–JAK3. §IL‐6 signals through JAK1–JAK2 or TYK2. ||IL‐12 and IL‐23 signal through JAK2–TYK2. ¶IFNα signals through JAK1–TYK2.

Although currently approved drugs have demonstrated varying degrees of selectivity for JAK isoform, the capacity of each JAK inhibitor to inhibit specific cytokine-signaling pathways is not readily predictable from preclinical selectivity data in direct cell-based assays. For instance, Dowty et al. compared the inhibitory effects of tofacitinib, baricitinib, upadacitinib, and filgotinib on cytokine-induced STAT phosphorylation patterns in whole blood cells at clinically efficacious doses, revealing generally similar cytokine receptor inhibition profiles with minor numerical differences (353). An additional *in vitro* pharmacological analysis examined the regulation of cytokine signaling by baricitinib, upadacitinib, and tofacitinib in human leukocyte subpopulations. While distinct cytokine pathways were modulated to varying degrees by different JAK inhibitors, no single agent potently or continuously inhibited an individual cytokine signaling pathway throughout the dosing interval (354). In an industry-sponsored study, filgotinib was found to inhibit JAK1-mediated signaling similarly to other JAK inhibitors but with reduced inhibition of JAK2-dependent and JAK3-dependent pathways (355). Moodley et al. assessed the *in vivo* impact of pan- and selective JAK inhibitors in mice through immunologic, genomic, and epigenomic profiling. While selective cell type-specific effects of JAK inhibitors were observable, there was a high overall overlap between these compounds (356). Clinically, no head-to-head trials have compared the efficacy of these agents; however, they have all demonstrated similar therapeutic effects in various autoimmune or immune-mediate diseases, such AR (357, 358), inflammatory bowel disease (351), AD (359) or AA (360). In summary, current experimental data do not conclusively indicate the potential advantages of higher selectivity in next-generation JAK inhibitors. It is only through rigorous clinical testing, including head-to-head studies and real-world application, that the clinical significance of differences between various JAK inhibitors will be determined.

## 7 Conclusions

In recent years, advancements in understanding cytokine biology and its regulatory interplay with autoimmunity have catalyzed a revolution in the treatment of autoimmune diseases. Despite this progress, the persistent burden of these conditions underscores the ongoing need for novel therapeutics. Small molecule targeted drugs, such as JAK inhibitors, capable of simultaneously blocking multiple cytokines signaling pathways, have demonstrated remarkable efficacy in a variety of autoimmune and immune-mediated inflammatory diseases, heralding a new era in treatment.

This review comprehensively summarizes the role of cytokines signaling through the JAK-STAT pathway in the pathogenesis of BP and PV, positing JAK inhibitors as a potential novel therapeutic approach for AIBD. However, the specific mechanisms of JAK/STAT in the pathogenesis and progression of AIBDs remain to be fully elucidated. The distinct roles of JAK1, JAK2, JAK3, and TYK2 in PV and BP remain unclear, necessitating further research to clarify their contributions to disease mechanisms. Additionally, the interplay between STAT-dependent and non-STAT-dependent pathways in cytokine signaling requires exploration, as they may differentially impact disease progression and treatment outcomes. The correlation between JAK-STAT activation and disease severity also remains poorly understood, highlighting the need for mechanistic studies to determine whether JAK inhibitors block autoantibody production or merely reduce inflammation.

In summary, despite the promising therapeutic prospects, challenges remain in optimizing the use of JAK inhibitors. Selectivity for different JAK isoforms, potential side effects, and long-term safety concerns are areas that require further investigation. Additionally, the identification of biomarkers to predict treatment response and the development of combination therapies to enhance efficacy and reduce toxicity are active areas of research. Currently, there is a lack of comprehensive data on these aspects, and future studies will be essential to fill these gaps.
